# Editorial: Plant food bioactives, genomics, and health effects

**DOI:** 10.3389/fnut.2023.1166149

**Published:** 2023-03-08

**Authors:** Irena Krga, Tatjana Ruskovska, Dragan Milenkovic

**Affiliations:** ^1^Department of Nutrition, University of California, Davis, Davis, CA, United States; ^2^Centre of Research Excellence in Nutrition and Metabolism, Institute for Medical Research, National Institute of Republic of Serbia, University of Belgrade, Belgrade, Serbia; ^3^Faculty of Medical Sciences, Goce Delcev University, Stip, North Macedonia

**Keywords:** plant food bioactives, polyphenols, nutrigenomics, mechanisms of action, biological effects, alkaloids, bioinformatics, health benefits

Different plant food compounds, such as polyphenols, carotenoids, alkaloids, and phytosterols, are widely recognized for their bioactivity and potential role in preventing or delaying the onsets of many chronic diseases, including cardiometabolic and neurodegenerative diseases and cancer (1–4). Various studies have shown the antiinflammatory, antioxidant, vasculoprotective, immunomodulatory, anticarcinogenic, and other biological effects of these bioactives. Still, the exact mechanisms underlying their actions are not fully understood. Emerging data suggest that these compounds exert their biological effects by affecting multiple molecular targets, mainly through different genomic and epigenetic mechanisms (5–8). They seem to act at various levels of cell regulation, most probably simultaneously, affecting DNA methylation and histone modification processes modulating the expression of both protein-coding and non-coding genes and protein expression and activity (9–13). However, studies employing holistic, untargeted approaches that do not only look at the selected targets are relatively rare, and therefore, understanding the overall complex mechanisms of actions of these compounds requires further investigation.

This Research Topic presents recent works examining the role of several plant food bioactives in the prevention and treatment of different chronic diseases by focusing on deciphering the complex molecular mechanisms of their actions. A review by Samota et al. provides a summary of the antidiabetic, antiobesity, cardioprotective, hepatoprotective, and anticancerous activities of anthocyanins, a polyphenol group of compounds regularly consumed in red-, purple- and violet-colored vegetables, fruits, and cereals. The authors also address the importance of factors influencing anthocyanin stability, including various extraction methods, in the bioavailability of these compounds and, ultimately, their biological effects. The multigenomic action of anthocyanin-rich extract from black beans on adipose tissue was demonstrated for the first time by Damián-Medina et al. in an animal model of type 2 diabetes. Using RNAseq and bioinformatic analyses, this study showed that anthocyanin-rich extract modulated the expression of both protein-coding and non-coding genes (miRNAs, lncRNAs, and snRNAs) that were identified as involved in regulating different pathways with notable roles in type 2 diabetes pathogenesis, such as insulin secretion, PI3K signaling, NIN/NF-κB signaling, and endoplasmic reticulum organization. More importantly, these nutrigenomic effects were paralleled by improved blood glucose and inflammatory marker levels, suggesting that the antidiabetic effects of this anthocyanin-rich extract are achieved through complex genomic regulation in the adipose tissue. Transcriptomic modifications in adipose and hepatic tissue were also observed following dietary supplementation with quercetin in the context of metabolic syndrome (Kábelová et al.). In this animal study, quercetin—which is abundantly present in apples, onions, berries, and grapes—was shown to affect the expression of different genes linked with the regulation of lipid and fatty acid metabolism and also the functions associated with metabolic syndrome. Moreover, the authors identified critical regulatory nodes, including PPARG, NOS2, ADIPOQ, and Mir378, and suggested their modulation by quercetin as the potential mechanism underpinning the reduced fat accumulation and improved glucose tolerance observed in animals that are fed a quercetin-enriched diet.

Besides the evidence from mechanistic animal studies, the pleiotropic mode of action of polyphenols was also reported in human subjects (Krga et al.). More specially, the consumption of flavanones in grapefruit juice for 6 months altered the expressions of numerous protein-coding genes and miRNAs in peripheral blood mononuclear cells of postmenopausal women. Moreover, this effect was paralleled by improvements in arterial stiffness, evaluated as pulse wave velocity. By combining various bioinformatic analyses, this study revealed the complex mechanisms underlying the vasculoprotective effects of grapefruit flavanones, including interactions with several transcription factors and cell signaling proteins and modulations in the expression of both mRNAs and miRNAs involved in inflammation, immune response, cell interaction, and motility. Altogether, this results in a global gene expression profile that is inversely correlated with those observed in hypertension and arterial stiffness.

In addition to polyphenols, other plant food bioactive compounds have also been evaluated for their biological effects and underlying mechanisms of actions, particularly in the context of exploring complementary and alternative therapeutic options to conventional drug treatments for chronic diseases. By employing *in vitro* studies, *in silico* molecular docking, and enzyme inhibition assessments, Hu et al. showed the potency of the oligopeptides VYGF, GLLGY, and HWP, obtained from fermented rice bran, to inhibit the angiotensin-converting enzyme, suggesting their potential use in managing hypertension. Moreover, they revealed that HWP could also inhibit the activity of pancreatic lipase and potentially be used in the control of obesity. On the other hand, alkaloid leonurine hydrochloride was identified by Wu et al. as a potent antioxidant and hepatoprotective agent *in vitro* that is promising for potential use against liver damage in alcoholic liver disease. By combining RNA-seq and metabolomic analyses, this study identified modulations of lipid metabolism, particularly glycerophospholipid metabolism, as an essential target through which this alkaloid could mediate its action against this liver disease.

In summary, the results from this Research Topic further support the use of different omics and bioinformatic approaches to tackle the complex, multilevel mode of action of plant food bioactives and provide an understanding of their effects. Future research should involve more multiomics explorations of the actions of these compounds in physiologically relevant conditions in both *in vitro* and *in vivo* studies, and particularly human trials in different disease contexts, to obtain a comprehensive picture of the molecular mechanisms underlying the health-promoting properties of these bioactives. Moreover, mechanistic explorations in subjects of varying ethnicity, gender, age, or genetic background will allow to explore the variability in responsiveness to plant food bioactives (14) and pave the way for precision nutrition practices.

## Author contributions

IK wrote and TR and DM reviewed the manuscript. All authors approved the final version of the manuscript.
